# Mechanisms of eukaryotic replisome disassembly

**DOI:** 10.1042/BST20190363

**Published:** 2020-06-03

**Authors:** Sara Priego Moreno, Agnieszka Gambus

**Affiliations:** 1 The Salk Institute for Biological Studies, 10010 N Torrey Pines Rd, La Jolla, CA 92037, U.S.A.; 2 Institute for Cancer and Genomic Sciences, College of Medical and Dental Sciences, University of Birmingham, Edgbaston, Birmingham B15 2TT, U.K.

**Keywords:** Cullin2-LRR1, genome integrity, p97, replisome disassembly, TRAIP, ubiquitylation

## Abstract

DNA replication is a complex process that needs to be executed accurately before cell division in order to maintain genome integrity. DNA replication is divided into three main stages: initiation, elongation and termination. One of the key events during initiation is the assembly of the replicative helicase at origins of replication, and this mechanism has been very well described over the last decades. In the last six years however, researchers have also focused on deciphering the molecular mechanisms underlying the disassembly of the replicative helicase during termination. Similar to replisome assembly, the mechanism of replisome disassembly is strictly regulated and well conserved throughout evolution, although its complexity increases in higher eukaryotes. While budding yeast rely on just one pathway for replisome disassembly in S phase, higher eukaryotes evolved an additional mitotic pathway over and above the default S phase specific pathway. Moreover, replisome disassembly has been recently found to be a key event prior to the repair of certain DNA lesions, such as under-replicated DNA in mitosis and inter-strand cross-links (ICLs) in S phase. Although replisome disassembly in human cells has not been characterised yet, they possess all of the factors involved in these pathways in model organisms, and de-regulation of many of them are known to contribute to tumorigenesis and other pathological conditions.

## Introduction

Genome replication is a highly regulated and co-ordinated process that our cells need to complete before undergoing cell division. Importantly, cells have evolved very sophisticated mechanisms that allow them to achieve this accurately and efficiently. However, it is known that factors such as genetic alterations and environmental hazards can promote defects in this process giving rise to mutations and genomic aberrations that can lead to cancer and other pathological diseases. Therefore, understanding in detail the mechanisms that underlie genome replication is crucial to apprehend the development of these human disorders.

**Figure 1.**
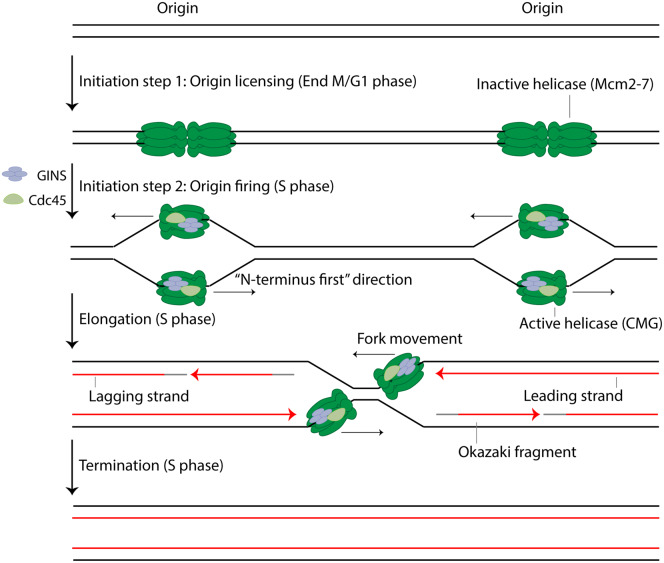
Simplified model of eukaryotic DNA replication. Inactive double hexamers of the replicative helicase core Mcm2–7 are loaded onto origins at the end of mitosis/G1 phase (origin licensing). Origins are activated during S phase with the assembly of the active replicative helicase, also known as CMG (Cdc45-Mcm2–7-GINS), which involves the recruitment of GINS and Cdc45 to the Mcm2–7 complex. While double hexamers of Mcm2–7 encircle dsDNA, CMGs encircle ssDNA and translocate in an ‘N-terminus first' direction thus passing each other within the origin (origin firing). Replisomes are built around CMGs and they progress through the chromatin unwinding DNA and promoting DNA synthesis in a semi-discontinuous way (elongation). Replication forks from neighbouring origins finally converge, leading to complete replication of sister chromatids (termination).

The process of DNA replication can be divided into three main stages: initiation, elongation and termination (Figure 1). The initiation stage involves the activation of origins of replication, which leads to the generation of two replication forks that move away from each other in opposite directions. During elongation, these replication forks progress through the chromatin unwinding and replicating DNA until two forks from neighbouring origins meet each other and the termination stage takes place. Termination involves completing synthesis of the stretches of DNA between converging forks and the disassembly of the replication machinery from fully replicated chromatin. While the molecular mechanisms taking place during initiation and elongation of DNA replication have been fairly well described over the last number of decades, little was known about the mechanisms underlying the termination stage. In the last six years however, biochemistry and cell biology research carried out using *Saccharomyces cerevisiae*, *Xenopus laevis* egg extract and *Caenorhabditis elegans* embryos has allowed us to draw the first model of the mechanism of eukaryotic DNA replication termination.

In this review, we give a general overview of the main events taking place during the initiation, elongation and termination of eukaryotic DNA replication, and describe in more detail the mechanism of replisome disassembly upon DNA replication termination. We also describe other situations in which disassembly of the replisome is required to maintain genome integrity.

## Overview of eukaryotic DNA replication

Chromosome replication starts from origins of replication, which are the genomic locations where loading and activation of the replicative helicases takes place resulting in double-stranded DNA (dsDNA) unwinding and exposure of single-stranded DNA (ssDNA) templates for DNA synthesis. Eukaryotic cells establish many origins of replication (around 50 000 in human cells) in every cell cycle that are stochastically activated throughout S phase following a strict replication timing programme [1]. Each chromosome must be replicated only once per cell cycle, as re-replication of genomic regions can be deleterious for the maintenance of genome integrity. This is achieved by the tight regulation of origin activation through a two-step mechanism separated into two different cell cycle stages [2]. The first step, also known as origin licensing, only takes place before the onset of S phase (end of mitosis/G1) when CDK activity is low, and it consists of the loading of double hexamers of the replicative helicase core Mcm2–7 onto origin DNA through the concerted action of the factors ORC, Cdc6 and Cdt1 [3,4]. The products of this reaction are enzymatically inactive double hexamers of Mcm2–7 encircling dsDNA in a head-to-head orientation, thus establishing already bidirectionality of DNA replication (Figure 1, Origin licensing). The second step of origin activation only occurs during S phase when the CDK activity is high, and it consists of the activation of the replicative helicase Mcm2–7 by the kinases DDK and CDK [5]. While DDK phosphorylates the Mcm2–7 complex and promotes recruitment of the accessory subunit Cdc45, recruitment of the GINS complex is dependent on both DDK and CDK activity. These events result in the assembly of the replicative helicase also known as the CMG complex (Cdc45-Mcm2–7-GINS). At each origin, two CMG complexes initiate DNA unwinding and reconfigure their mode of DNA binding by encircling now only ssDNA [6]. The activity of Mcm10 leads to further DNA untwisting and helicase activation, followed by translocation of the helicases on opposing DNA strands in an ‘N-terminus first' direction, thus passing each other within the origin (Figure 1, Origin firing). CDK activity during S phase also inhibits origin licensing by compromising the stability of some licensing factors. This is one of the main mechanisms by which cells avoid re-replication [7].

During elongation of DNA replication, two replication forks emerge from each activated origin as the replisome machinery assembles around each individual CMG. Replication fork progression through the chromatin is, in large part, facilitated by the CMG complex, which sits at the tip of the replication fork and unwinds dsDNA by translocating along the leading strand template with 3′–5′ polarity [8]. This dsDNA unwinding generates ssDNA, which provides the templates required for DNA synthesis by the replicative polymerases. Because of the antiparallel structure of the DNA and the 3′ end extension specificity of the replicative polymerases, DNA replication proceeds in a semi-discontinuous way: the leading strand is copied continuously in the direction of fork progression, while the lagging strand is copied discontinuously in the opposite direction via short Okazaki fragments that need to be processed and ligated in order to generate a complete DNA strand (Figure 1, Elongation) [9]. Several processes are coupled to replication fork progression during elongation, including: the topoisomerase-dependent release of torsional stress generated ahead of the fork as a consequence of DNA unwinding [10], the establishment of sister chromatid cohesion [11] and the re-establishment of chromatin structure and epigenetic marks in the newly synthesised DNA [12].

**Figure 2.**
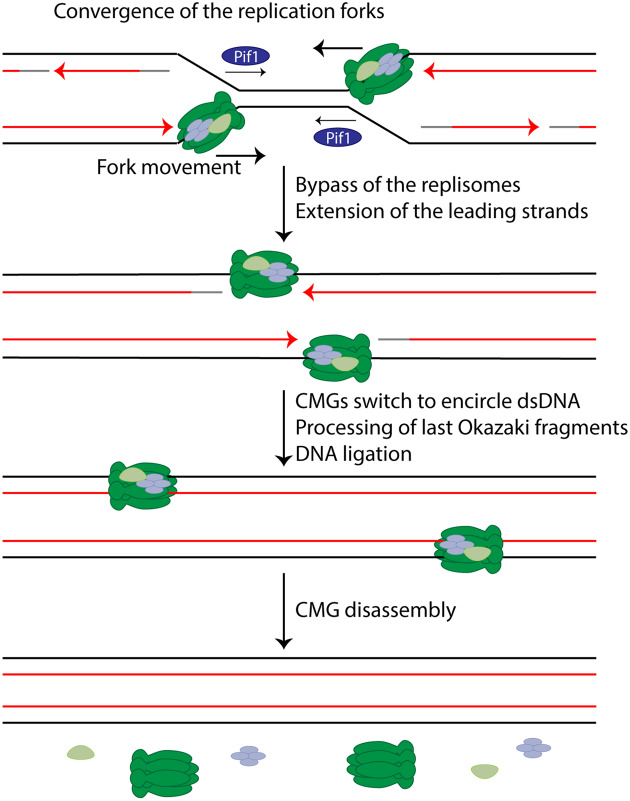
Mechanism of eukaryotic DNA replication termination. Convergence of replication forks during termination is facilitated by Pif1 family helicases, likely due to their ability to unwind DNA in the opposite direction of CMG movement. CMGs continue to translocate on the leading strand templates and pass one another while leading strands continue to be extended. CMGs encounter the last Okazaki fragments on lagging strands and switch to encircle dsDNA. The last Okazaki fragments are processed by an unknown mechanism. CMGs are disassembled only after DNA has been fully ligated.

Finally, termination of replication forks occurs when two forks arising from neighbouring origins converge between two replicons (Figure 1, Termination). In eukaryotic cells, termination of replication forks occurs throughout the entire S phase, whenever and wherever two replication forks converge [13,14]. One of the hallmark studies that shed light into the mechanism of eukaryotic DNA replication termination came from the Walter lab [15]. In order to study termination, they constructed plasmids with a reversible replication fork barrier consisting of an array of *lac* operators (LacOs) that could bind *lac* repressors (LacRs). Incubation of these vectors in *Xenopus* egg extract containing LacR led to the replication of most of the vector DNA and forks stalling at the edges of the LacR array. Upon addition of IPTG to the extract, the LacR array was disrupted, allowing synchronous and site-specific termination of replication forks. Using this system, it has been shown that DNA synthesis within the array did not slow down during convergence of replication forks, and nascent leading strands were extended until they encountered the last Okazaki fragment of the downstream lagging strand with replisomes passing each other (Figure 2). The fact that the CMG helicase encircles the leading strand template means that converging replisomes approach each other on opposite strands, which helps us to understand how they can pass one another at termination, as well as during origin activation [6]. Finally, daughter strands were fully ligated, replisomes removed from the DNA and sister chromatids decatenated by topoisomerase II. The fact that replisome disassembly in this system could happen after DNA molecules were fully ligated suggests that the replisomes are disassembled from dsDNA and that replisome unloading is the last stage of replication termination. Termination of replication forks has also been reconstituted *in vitro* with purified budding yeast proteins by the Labib group [16]. The data shown in this study indicates that reconstituted replisomes stall upon convergence, resulting in deficient termination of replication forks and the resultant accumulation of late replication intermediates. This termination defect was rescued upon addition of either of the 5′-3′ polarity Pif1 family helicases Rrm3 and Pif1, indicating that convergence of eukaryotic replisomes requires the activity of these helicases; most likely they help to finish unwinding the last stretches of parental DNA (Figure 2). This termination defect was also recapitulated *in vivo* during plasmid replication in budding yeast cells lacking both Rrm3 and Pif1 [16]. In this case, fork convergence was delayed but not abolished, suggesting that other redundant pathways must exist to promote replisome convergence. Further hallmark studies published within the last few years have added important pieces to the puzzle of DNA replication termination by elucidating the mechanism by which the replisome is disassembled. These are discussed in the next sections.

## Mechanisms of eukaryotic replisome disassembly

While replisome assembly during initiation of DNA replication has been studied and described in detail, the mechanism of replisome disassembly remained elusive until a few years ago. Since the Mcm2–7 complex cannot be loaded or re-loaded during S phase, its disassembly from chromatin needs to be restricted to the point at which its job is complete i.e. when the fragment of DNA between two origins is fully replicated. Interestingly, recent research has demonstrated that replisome disassembly is also triggered in response to specific forms of DNA damage.

### Replisome disassembly upon termination of replication forks

**Figure 3.**
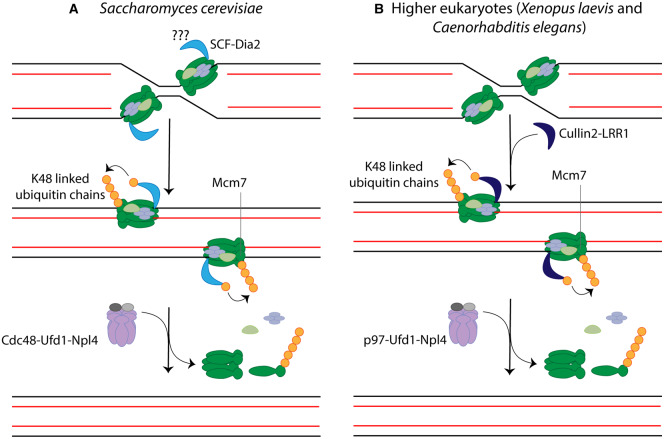
Mechanisms of eukaryotic replisome disassembly during termination. (**A**) Replisome disassembly during termination in *Saccharomyces cerevisiae*. SCF^Dia2^ might be constitutively associated with the budding yeast replisome and promotes CMG ubiquitylation on its Mcm7 subunit during termination in the form of K48-linked ubiquitin chains. Alternatively, it is possible that SCF^Dia2^ is recruited to the replisome only during termination, leading to Mcm7 ubiquitylation. CMG ubiquitylation triggers Cdc48-Ufd1-Npl4-dependent recruitment and replisome disassembly. (**B**) Replisome disassembly during termination in higher eukaryotes. DNA replication termination promotes *de novo* recruitment of Cullin2^LRR1^ to the terminated CMGs, which results in the ubiquitylation of Mcm7 with K48-linked ubiquitin chains. The ubiquitylated replisome is subsequently disassembled by p97-Ufd1-Npl4.

Post-translational modifications are essential in almost every cellular process, including DNA replication, and this is due to their ability to dynamically regulate protein behaviour. For instance, assembly of the CMG helicase onto chromatin during initiation of DNA replication requires CDK and DDK mediated phosphorylation of the Mcm2–7 complex and other initiator factors [7]. Instead, the mechanism of replisome disassembly during termination was shown to be strictly regulated by ubiquitylation of the Mcm7 subunit of the CMG helicase [17,18]. Work from the Labib and the Gambus groups has shown that during DNA replication termination in S phase, replisomes are modified with lysine 48 (K48)-linked ubiquitin chains on the Mcm7 subunit of the CMG helicase, which leads to replisome disassembly [17,18]. This ubiquitylation is promoted by the Cullin/Ring Ubiquitin Ligases (CRL) Cullin2^LRR1^ in metazoans [19,20] and Skp1-Cullin1-F-box protein complex SCF^Dia2^ in budding yeast (Figure 3) [18]. For further understanding of CRLs modular composition and ubiquitylation we recommend the reader to access one of our previous reviews [21].

A fundamental, but still not answered question is how Mcm7 ubiquitylation is restricted to termination. It is likely that the CMG complex is remodelled during DNA replication termination leading to the exposure of specific protein domains required for the recruitment of the ubiquitin ligase to the replisome and/or for the ubiquitylation of Mcm7. Such recruitment of the ligase to the replisome has been studied in budding yeast and in *Xenopus* egg extracts. The association between SCF^Dia2^ and the replisome has been well characterised in extracts made with budding yeast cells in which proteins were released from chromatin by DNA digestion [22–24]. In these extracts, SCF^Dia2^ associates with the replisome *via* multiple interactions mediated by both the N-terminal tetratricopeptide repeat (TPR) and the C-terminal leucine-rich repeats (LRR) domains of Dia2 [23,24]. The TPR domain of Dia2 tethers the ligase to the replisome *via* interaction with the replisome subunits Mrc1 (yeast homologue of Claspin) and Ctf4 (AND-1 in higher eukaryotes) [23]. The LRR domain, which is thought to be the substrate recognising domain of Dia2, could be promoting the association with the replisome by directly interacting with the Mcm7 subunit of the CMG complex, although this still needs to be demonstrated. Moreover, while the efficiency of Mcm7 ubiquitylation and CMG disassembly are reduced upon disruption of the TPR-dependent tethering of SCF^Dia2^ to the replisome [25], it remains to be determined how these processes are affected upon disruption of the LRR-dependent interaction. The interaction between SCF^Dia2^ and the replisome peaked in S phase extracts and was preserved in extracts made with cells that had been treated with hydroxyurea, which led to the early conclusion that the ligase associated constitutively with the replisome at replication forks [23]. However, this conclusion was questioned in a later study when researchers observed that all of the Mcm7 molecules within CMGs, including those that were present at active replication forks, were very efficient substrates for ubiquitylation after nuclease treatment [18]. This could mean that release of replisomes from DNA by DNA digestion mimics events of termination leading to recruitment of the ligase to the replisome and efficient Mcm7 ubiquitylation. Alternatively, the ligase might be constitutively associated with the budding yeast replisome, perhaps through the TPR-dependent interaction, but remodelling of the CMG complex upon termination (and after DNA digestion) allows for the recognition of the Mcm7 subunit by the LRR domain and ubiquitylation of Mcm7. Therefore, despite the well characterised interaction between SCF^Dia2^ and the replisome, the timing of SCF^Dia2^ recruitment to the yeast replisome during DNA replication is still an open issue to be resolved in the future. In contrast with Dia2, LRR1 does not have a TPR domain, and it most likely interacts with the CMG complex through its LRR domain, although this has not been experimentally demonstrated yet. However, although our understanding in regards to the domains required for the LRR1 association with the replisome is much more limited than in the case of Dia2, we understand better the regulation of the timing when Cullin2^LRR1^ associates with the replisome during DNA replication. Work in *Xenopus* egg extract has shown that Cullin2^LRR1^ is only recruited to the replisome upon termination, as inhibition of fork convergence prevents association of the ligase with the chromatin bound replisome and compromises Mcm7 ubiquitylation [19,20]. Therefore, it seems that for Cullin2^LRR1^ Mcm7 ubiquitylation is controlled by the regulated recruitment of the ligase to the replisome during DNA replication termination. However, the signal that promotes the recruitment of the ligase is still unknown, but it is likely to involve remodelling of the CMG complex during termination as mentioned above.

Another important aspect that is still poorly understood is whether other enzymatic activities are required for the ubiquitylation of Mcm7. It has been shown that protein ubiquitylation by CRLs sometimes requires the function of a member of the Ariadne family of RBR ubiquitin ligases, which add the first ubiquitin to the substrate, hence priming it for further ubiquitylation by the CRL enzyme [26,27]. However, work in budding yeast has shown that Mcm7 ubiquitylation is not affected by depletion of RBR or HECT ubiquitin ligases [24]. Moreover, Mcm7 ubiquitylation has been reconstituted *in vitro* with a protein complex of replisome-SCF^Dia2^ purified from budding yeast without the need to include other ubiquitin ligases in the reaction [24]. Nevertheless, since the ubiquitin ligase is different in higher eukaryotes, we cannot rule out that other enzymatic activities are involved in priming Mcm7 for Cullin2^LRR1^ driven ubiquitylation.

Once ubiquitylated upon termination, the replisome is recognised and disassembled by the p97 segregase, which results in the clearance of terminated CMGs from chromatin (Figure 3) [15,17,18]. p97, also known as VCP in human cells or Cdc48 in budding yeast, is a ring-shaped homo-hexameric and highly conserved AAA^+^ ATPase [28]. It works as a ubiquitin specific segregase by binding to ubiquitylated proteins and extracting them from a variety of stable cellular structures including protein complexes, membranes and chromatin. In order to provide substrate specificity, p97 associates with a large variety of cofactors that also contain ubiquitin binding modules and aid p97 to recognise specific substrates. For example, the heterodimeric cofactor Ufd1-Npl4 is very well known to assist p97 in the unloading of many chromatin-associated proteins [29–31]. Disassembly of the ubiquitylated CMG has been shown to require the cofactor Ufd1-Npl4 *in vivo* in budding yeast [32] and metazoans [19,33]. Moreover, the replisome disassembly reaction has successfully been reconstituted *in vitro* with budding yeast pulled down replisomes and purified segregase proteins revealing that p97, Npl4 and Ufd1 are sufficient to promote disassembly of the previously ubiquitylated replisome [24]. At present we do not know whether other p97 secondary cofactors play a role during the CMG disassembly reaction in higher eukaryotes.

Two recent studies have shed light onto the mechanism by which p97 unfolds its substrates by determining the active conformation structure of Cdc48 in complex with a substrate-recruiting cofactor and a polyubiquitylated substrate by cryo-electron microscopy (cryo-EM) [34,35]. These reconstructed structures imply a hand-over-hand mechanism of translocation for p97 similar to that observed for other AAA^+^ ATPases [36].The structures obtained for Cdc48 in complex with Ufd1-Npl4 revealed that this complex starts to process its substrates by binding and unfolding a distal ubiquitin molecule within the substrate attached chain [35]. The unfolded ubiquitin is then projected through the ATPase rings of Cdc48, followed by translocation of the remaining ubiquitin chain and the substrate polypeptide through the central channel of the segregase, finally leading to the release of the unfolded oligo-ubiquitylated polypeptide. This mechanism explains why p97/Cdc48 works best with shorter ubiquitin chains (about 5 ubiquitin monomers), as they do not require prior unwinding of extended ubiquitin chains before processing the substrate itself. The defined pattern of modifications over Mcm7 observed by immunoblotting in budding yeast and *Xenopus* egg extracts, suggest restricted and regulated levels of ubiquitylation [17,18,24,32]. Altogether, it is tempting to speculate that the level of Mcm7 ubiquitylation is restrained to streamline p97/Cdc48 extraction. We can imagine, therefore, that ubiquitylated Mcm7 may be translocated through the central channel of Cdc48/p97, which leads to dissociation of the different CMG components [19,24,32]. However, at present we do not know whether Mcm7 and the other CMG subunits are unfolded during the disassembly reaction.

A critical role for p97 is to induce the initial unfolding of ubiquitylated substrates in order to prepare them for subsequent proteasomal degradation [37]. While the fate of ubiquitylated Mcm7 and the rest of the CMG complex after disassembly is still unknown, one study did find that pharmacological inhibition of proteasomal degradation in *Xenopus* egg extract only led to a minor accumulation of ubiquitylated Mcm7 in comparison to control extracts [38]. These data suggest that, at least in *Xenopus* egg extract, the proteasome plays a minor or redundant role in the turnover of ubiquitylated Mcm7 following CMG disassembly. Future work is needed to reinforce this data and to determine the fate of the different subunits of the CMG complex upon disassembly.

Finally, although Mcm7 is the only CMG subunit that has been found ubiquitylated during DNA replication termination [17,18], we still lack direct evidence supporting that the ubiquitylation of Mcm7 is essential for CMG disassembly. Work in budding yeast has tried to address this issue by mapping the lysine (K) residues ubiquitylated within Mcm7 and validating their importance for Mcm7 ubiquitylation and CMG disassembly [32]. The N-terminal K29 was the major site of Mcm7 ubiquitylation both *in vivo* as well as *in vitro* in yeast extracts. However, while mutation of this K residue abrogated completely the *in vitro* ubiquitylation of Mcm7 in yeast extracts, the same Mcm7 mutant could be efficiently ubiquitylated on another site (or sites) and CMG disassembled *in vivo* [32]. An explanation for this could be that while K29 seems to be the only possible ubiquitin acceptor residue in the observed *in vitro* ubiquitylation, Mcm7 is always ubiquitylated on K29 *in vivo* but other K residues can become ubiquitin acceptors upon mutation of K29. Alternatively, it could be that the mass spectrometry assay performed in this study was unable to detect other K residues that are also ubiquitylated *in vivo*, but not *in vitro*, together with K29. In agreement with an important role for the N-terminal region of Mcm7 in the ubiquitylation of the protein, mass spectrometry analysis has revealed the residues K27 and K28 as the major ubiquitin acceptor sites within Mcm7 in S phase in *Xenopus* egg extract (unpublished data from the Gambus lab). However, these mutants of Mcm7 are yet to be validated and one can envisage similar problems as in budding yeast. Altogether, the generation of Mcm7 mutants unable to undergo ubiquitylation is a difficult task that will require further work.

### Other pathways triggering replisome disassembly

**Figure 4.**
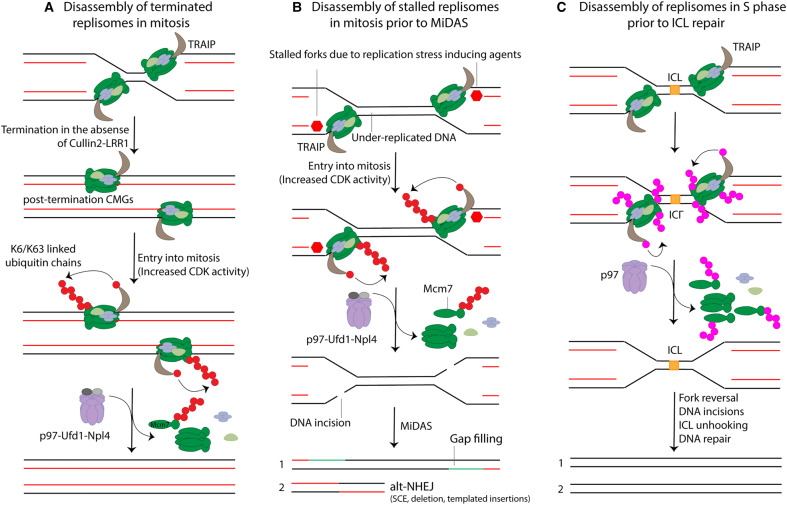
TRAIP-dependent replisome disassembly pathways. (**A**) Disassembly of terminated replisomes in mitosis. When termination occurs in the absence of Cullin2^LRR1^ activity, the terminated replisomes remain associated with chromatin during S phase. Entry into mitosis, driven by an increase in CDK activity, promotes TRAIP-dependent ubiquitylation of Mcm7 within terminated CMGs with K6/K63-linked ubiquitin chains. The ubiquitylated terminated replisome is finally disassembled by p97-Ufd1-Npl4. (**B**) Disassembly of stalled replisomes in mitosis required for the processing of under-replicated DNA. When DNA replication proceeds in the presence of replication stress-inducing agents such as aphidicolin, replication forks slow down and compromise forks convergence during S phase, thus increasing the risk of entering mitosis with under-replicated DNA. When mitosis ensues in the presence of under-replicated DNA, TRAIP promotes ubiquitylation of Mcm7 within stalled CMGs with long ubiquitin chains, followed by disassembly of the stalled replisomes by p97-Ufd1-Npl4 and DNA incisions in the leading strand templates. In this scenario, one of the chromatids is faithfully repaired through gap filling (1) while repair of the other one requires the joining of the two broken ends likely through the alt-NHEJ mechanisms of SSA and MMEJ. This chromatid, therefore, receives a deletion encompassing the under-replicated DNA and exhibits sister chromatid exchange (SCE) and potential templated insertions (2). (**C**) Disassembly of replisomes prior to ICL repair. Convergence of replication forks at both sites of the ICL triggers ubiquitylation of several CMG components by TRAIP. These ubiquitin chains are long and heterotypically linked and/or branched. Ubiquitylated CMGs are disassembled by p97, followed by fork reversal, nucleolytic incisions, ICL unhooking and DNA repair. One of the chromatids is repaired through translesion synthesis (1) while the other one is repaired through homologous recombination (2).

Budding yeast cells lacking Dia2 (*dia2*Δ cells) retain replisomes on chromatin through mitosis and into G1 of the next cell cycle [18], suggesting that CMG disassembly in budding yeast is controlled by only one pathway. However, this is not the case in higher eukaryotes, where recent work has revealed the existence of Cullin2^LRR1^-independent pathways for CMG disassembly. It has been shown that in the absence of Cullin2^LRR1^ activity the terminated replisomes remain on chromatin during S phase, but entry into mitosis leads to rapid replisome disassembly, indicating that a mitotic specific pathway is activated (Figure 4A) [19,39,40]. In this pathway, the ubiquitin ligase TRAIP is responsible for Mcm7 ubiquitylation and CMG disassembly [39,40]. Unlike in S phase, the ubiquitin linkages required for the mitotic pathway of replisome disassembly involves lysine 6 (K6) and lysine 63 (K63), and overall the ubiquitylation pattern is different, consistent with longer chains assembled on Mcm7 in mitosis [40]. At present we do not know whether this pathway requires ubiquitylation of other CMG components. Unlike Cullin2^LRR1^, TRAIP is constitutively associated with the replisome [20,41] and its activation during mitosis seems to be regulated by a mechanism that does not involve its *de novo* recruitment to the replisome. This mitotic pathway of replisome disassembly also requires p97 activity [39,40] and other factors, which were identified in *C. elegans* embryos, including the p97 cofactors UFD-1-NPL-4 and UBXN-3 (worm homologue of the vertebrate tumour suppressor Faf1), as well as the SUMO protease ULP-4 (worm homologue of the vertebrate SUMO protease SENP6/7) [19]. The exact roles of UBXN-3 and ULP-4 in mitotic replisome disassembly are yet to be elucidated. It would not be surprising, however, if yet more cofactors of p97 are also found to be required for this mitotic pathway as it seems likely that differing cofactors would aid recognition of the differing ubiquitin linkages. It will be interesting to investigate whether Faf1, as in *C. elegans*, plays any role in mitotic replisome disassembly in other metazoans. The role of the SUMO pathway during mitotic replisome disassembly has been studied in *Xenopus* egg extract and it has been shown that the latter is not affected by enhanced SUMO conjugation or inhibition of SUMO deconjugation. Moreover, inhibition of SUMO conjugation did not affect mitotic replisome disassembly either, indicating that the activity of the SUMO pathway is not essential in replisome disassembly during mitosis in this model system [40]. Therefore, the involvement of ULP-4 during mitotic replisome disassembly in *C. elegans* might be restricted to this organism, or not recapitulated in *Xenopus* egg extract, or its enzymatic activity is simply not required for mitotic replisome disassembly; instead, it could mediate protein–protein interactions.

Strikingly, it has been shown that during mitosis it is not only the terminated replisomes that are unloaded in a TRAIP-dependent manner, but also those that are stalled and were not able to complete replication due to induction of replication stress in the previous S phase (Figure 4B) [39,40,42]. Data obtained in *Xenopus* egg extract show that TRAIP-dependent disassembly of stalled replisomes in mitosis causes fork breakage and repair, likely through the alternative non-homologous end joining (alt-NHEJ) mechanisms of single strand annealing (SSA) and microhomology-mediated end joining (MMEJ) [39]. This, in turn, leads to localised genomic alterations such as deletions and templated insertions, which are characteristic of SSA and MMEJ mechanisms, respectively [43]. The authors proposed a model in which when replication forks stall before completing DNA replication, entry into mitosis triggers CMG disassembly by TRAIP, leading to the exposure and breakage of the leading strand templates. In this scenario, one of the sister chromatids would remain intact after being repaired through gap filling while the other one would be restored through alt-NHEJ of the two broken ends and would receive a small deletion encompassing the under-replicated locus. Interestingly, the chromosomal alterations observed as a result of this breakage and repair programme triggered by TRAIP are similar to those observed at human common fragile sites (CFSs): high frequency of sister chromatid exchanges, microhomologies at the breakpoint junction and small deletions [39,44]. Therefore, it is likely that the mechanism of mitotic replisome disassembly is important to maintain the integrity of CFSs. In agreement with a role of TRAIP in mitotic DNA repair, work in human cells has shown that the ubiquitin ligase activity of TRAIP is required for mitotic DNA synthesis (MiDAS) in cells that enter mitosis with incomplete replicated DNA [42]. Together these data suggest that disassembly of stalled replisomes in mitosis by TRAIP is a prerequisite for the processing of under-replicated DNA prior to cell division.

Recent work has also shown that the TRAIP induced replisome disassembly pathway is particularly important as a prior step required for the repair of certain types of DNA damage during S phase. Specifically, TRAIP has been shown to have a role in the repair of ICLs by the Walter group (Figure 4C). One of the main ways to repair ICLs is through the Fanconi Anemia (FA) pathway, which has been studied in detail in *Xenopus* egg extract. This FA pathway is initiated by convergence of replication forks at both sides of the ICL [45,46], which leads to the ubiquitylation of Mcm7 by TRAIP [47] and subsequent replisome disassembly by p97 [38]. In this scenario, other components of the CMG helicase including Mcm2, Mcm3, Mcm4, Mcm6 and Cdc45 are ubiquitylated in addition to Mcm7. Analogous to its activity during mitosis, the chains deposited by TRAIP on CMG during ICL repair seem to be long and heterotypically linked and/or branched [47]. CMG unloading by this mechanism enables fork reversal [48] and ICL unhooking through nucleolytic incisions [49,50] that convert the ICLs into double strand breaks, which are finally repaired through homologous recombination [51].

## Importance of replisome disassembly pathways in the maintenance of genome integrity

The fact that different pathways have evolved to ensure that all replisomes are disassembled prior to cell division suggests that it must be a very important event. One can envisage that both: (i) the uncontrolled unloading of the CMG at the wrong time and place and (ii) the failure to unload CMGs when needed, can be very detrimental to the maintenance of genome integrity.

Uncontrolled replisome unloading during S phase would be disastrous for genome stability and cell viability as the mechanisms to ensure once-per-cell-cycle DNA replication inhibit the re-loading of the helicase onto already established replication forks. Indeed, a critical role of the S phase checkpoint mechanisms is to safeguard the stability of the replisome at stalled forks allowing for its disassembly only when it is essential for the repair of the insult [52]. Therefore, we speculate that the existence of two different pathways for replisome disassembly in S phase allows cells to control the process at the maximum level: protect the active replisomes from unloading while efficiently disassembling terminated ones. On one hand, the TRAIP pathway would only be activated in situations in which replisome disassembly is essential for the repair of a certain lesion (i.e. ICLs). In this pathway, where the ligase is constitutively associated with the replisome, regulation of the ligase must be tightly controlled to avoid unscheduled replisome disassembly. TRAIP might have a way to switch between active and inactive conformation, perhaps through a post-translational modification or a conformational change in response to the DNA lesion. The activation of TRAIP may require a signalling cascade and not be instantaneous. On the other hand, the Cullin2^LRR1^ pathway would target specifically terminated replisomes during S phase, and its regulation would depend on its *de novo* recruitment to the terminated replisome as soon as replication forks converge and the Cullin2^LRR1^ interaction site is exposed. In mitosis, cells do not need to protect replisomes on chromatin. As mitosis ensues, TRAIP is activated, perhaps by mitotic signalling, and can trigger the unloading of both stalled and terminated replisomes. Deregulation of the fine balance between the pathways could be very detrimental and even moderate levels of TRAIP overexpression were reported to be cytotoxic [41]. However, whether this effect of TRAIP overexpression is due to uncontrolled replisome disassembly is unclear. Future work is needed to understand how overexpression or overactivation of Cullin2^LRR1^ may affect genomic stability.

Several studies have attempted to understand the consequences of blocking the process of CMG disassembly in different organisms. In budding yeast cells, absence of SCF^Dia2^ activity by deletion of DIA2 completely abrogates CMG disassembly. Interestingly, *dia2Δ* cells present several phenotypes suggesting that the protein plays a critical role in the maintenance of genome integrity. These cells are defective in cell cycle progression, present high levels of genomic instability, are unable to grow at low temperatures and are sensitive to drugs that compromise replication fork progression [23,53–55]. They also show genetic synthetic lethality in combination with other factors involved in DNA replication and repair [54,55]. However, whether these phenotypes are due to disruption of CMG disassembly or other unknown roles of the ligase in the maintenance of genome integrity is unclear. The best way to test this would be to see if the phenotypes observed upon deletion of DIA2 are recapitulated when CMG disassembly is inhibited by directly mutating the lysine residues within Mcm7 required for CMG ubiquitylation. However, as mentioned above, the mapping and mutation of these residues present several problems that have not been overcome yet. Therefore, further work is needed to understand to what extent the CMG disassembly pathway contributes to the maintenance of genome integrity in budding yeast. The consequences of disrupting the replisome disassembly pathways *in vivo* have also been studied in *C. elegans* embryos. While partial disruption of the S phase or mitotic pathways of replisome disassembly alone had no effect on worm embryo viability, disrupting both pathways led to embryonic lethality [19].

Recent work suggests that the mitotic pathway of replisome disassembly could be a key event to ensure that the presence of under-replicated DNA does not prevent chromosome segregation and cell division [42]. Although traditionally it was thought that DNA replication was completed and restricted to the S phase of the cell cycle, we now know that this is not always the case, especially when cells experience replication stress. To overcome replication stress, cells stop origin firing in new replicons [56] and sometimes slow down fork progression [57,58], and although this is important to avoid severe DNA damage, it also increases the risk of preventing convergence of replication forks and thus carrying under-replicated loci through mitosis. This scenario often occurs at CFSs, which are difficult to replicate and late-replicating genomic regions with poor origin density [44]. Under-replicated loci can be rescued during mitosis through the breakage and repair mechanism of MiDAS [59]. Under-replicated loci that escape MiDAS form ultrafine anaphase bridges (UFBs), which are dangerous structures that can result in micronuclei formation and non-disjunction of sister chromatids [60,61]. Upon dissolution of UFBs and chromosome segregation, the daughter cells inherit the under-replicated DNA, which is coated by 53BP1 protein in G1 of the next cell cycle [59,62,63] and still have a second chance to be repaired in the upcoming S phase [64]. Consistent with the idea of TRAIP-dependent replisome disassembly being required for MiDAS, it has been found that depletion of TRAIP in U2OS cells exposed to replication stress led to detection of many of the chromosomal instabilities observed when cells go through cell division with under-replicated DNA, including UFBs, micronuclei and 53BP1 bodies [42]. Although the programmed mechanism of breakage and repair induced by TRAIP during mitosis is accompanied by localised genome rearrangements [39], these instabilities are far less toxic than those arising from uncontrolled breakage during chromosome segregation in the presence of under-replicated DNA. As a result, at the human organism level, the reduction in TRAIP ubiquitin ligase activity, through mutation within the RING ubiquitin ligase active site, leads to microcephalic primordial dwarfism [65].

On a final note, efficient replisome disassembly before cell division is likely to be important also to avoid the accumulation of protein complexes on chromatin that could interfere with transcription and other DNA metabolic processes in the upcoming cell cycle. This aspect of the replisome disassembly defect still remains to be investigated.

## Outstanding questions

1. How are the ligases, involved in replisome disassembly, targeted or activated to ubiquitylate Mcm7 only at the right time? Cullin2^LRR1^ binds specifically to terminated helicase [19,20], but the mechanism by which it is targeted to this form of CMG helicase remains elusive. Are additional post-translational modifications involved? Does CMG change its conformation upon termination, or does its interaction with DNA change, to create new interaction platforms allowing for Cullin2^LRR1^ binding? On the other hand, TRAIP interacts with replisomes at all times [20,41] and thus what we need to understand in this case is the mechanism by which it is activated to ubiquitylate Mcm7 specifically at ICLs during S phase or in mitosis. Finally, although we know which parts of SCF^Dia2^ are required for its tethering to replisome [23,24], we still do not know whether it is recruited to terminated CMG *de novo* or if it interacts with the replisome constitutively and is then activated specifically at termination.
2. At present we do not know whether Mcm7 ubiquitylation is necessary or sufficient for replisome disassembly. The nature of ubiquitylation in its ability to modify different acceptor lysines, when the preferred ones are mutated, has perturbed the creation of an Mcm7 mutant that cannot be ubiquitylated, as described above. It is possible that a better understanding of the interaction between Mcm7 and SCF^Dia2^ or Cullin2^LRR1^ will allow us to create a mutant of Mcm7 that cannot support its ubiquitylation, while remaining fully active during other stages of replication.
3. The lack of an Mcm7 mutant that cannot be ubiquitylated prevents us also from determining the consequences of specifically blocking replisome disassembly. Deletion, depletion or down-regulation of the ubiquitin ligases involved in this process affect equally all of their substrates; it is, therefore, impossible to decipher the direct effects of replisome disassembly using these scenarios. Better approaches in targeting replisome disassembly will be required in the future to answer this question.
4. Another interesting point is to determine how the p97 segregase recognises ubiquitylated Mcm7 when it is modified by different types of ubiquitin chains during different scenarios i.e. K48 chains at termination [17,18], K6/63 chains in mitosis [40], mixed/branched chains at ICLs [47]. It is likely that a specific configuration of p97 cofactors is engaged in each of these situations.

## Perspectives

- The last six years of research have brought a rapid development of our understanding of the mechanisms by which the replisome is unloaded in different scenarios (i.e. during DNA replication termination, during mitosis to overcome under-replicated DNA, and during ICL repair). The fact that these mechanisms are strictly regulated throughout the cell cycle to ensure that replisome disassembly only occurs at the right place and at the right time suggests that deregulation of these mechanisms could be detrimental for the maintenance of genome integrity and have a negative impact on human health.
- Although we have learnt a lot about different mechanisms for replisome disassembly in the last 6 years, these mechanisms require further characterisation and several outstanding questions discussed in the previous section need to be answered in the future. Moreover, it is likely that replisome disassembly is also a pre-requisite for the repair of other forms of DNA damage different to the ones explained in this review. Therefore, we can only expect that our knowledge about replisome disassembly mechanisms will continue to expand in the next years.
- All proteins identified in the pathways of replisome disassembly described above, in studies using model organisms, are conserved in human cells. Both LRR1 and TRAIP are essential for cell viability and TRAIP has been shown to be important for the maintenance of genome stability in human cells [41,65–67]. However, it remains to be demonstrated whether they are responsible for ubiquitylation of Mcm7 and unloading of the replisomes also in human cells. As such, we do not know if it is their role in replisome disassembly that is essential for supporting cell viability or whether this is due to their role in modifying other substrates.

## Abbreviations

CFSs: common fragile sites
CMG: Cdc45-Mcm2–7-GINS
CRL: Cullin/Ring Ubiquitin Ligases
FA: Fanconi Anemia
ICLs: inter-strand cross-links
LRR: leucine-rich repeats
MMEJ: microhomology-mediated end joining
MiDAS: mitotic DNA synthesis
SSA: single strand annealing
TPR: tetratricopeptide repeat
UFBs: ultrafine anaphase bridges
